# Ru(bpy)_3_^2+^/nanoporous silver-based electrochemiluminescence immunosensor for alpha fetoprotein enhanced by gold nanoparticles decorated black carbon intercalated reduced graphene oxide

**DOI:** 10.1038/srep20348

**Published:** 2016-02-01

**Authors:** Wenjuan Zhu, Xiaohui Lv, Qi Wang, Hongmin Ma, Dan Wu, Tao Yan, Lihua Hu, Bin Du, Qin Wei

**Affiliations:** 1 Key Laboratory of Chemical Sensing & Analysis in Universities of Shandong, School of Chemistry and Chemical Engineering, University of Jinan, Jinan 250022, P.R. China; 2 School of Material Science and Engineering, University of Jinan, Jinan 250022, P.R. China

## Abstract

A highly sensitive sandwich-type electrochemiluminescence (ECL) immunosensor was proposed for the quantitative determination of alpha fetoprotein (AFP) using gold nanoparticles decorated black carbon intercalated reduced graphene oxide (Au-rGO@CB) as sensing platform and nanoporous silver (NPS) loaded Ru(bpy)_3_^2+^ as labels. In this work, intercalation of CB inhibited the accumulation of rGO and Au-rGO@CB was firstly used to immobilize primary antibody (Ab_1_) in ECL system. NPS prepared by the dealloying of binary alloy has high pore volume and surface areas, which was used to load amount of secondary antibodies (Ab_2_) and Ru(bpy)_3_^2+^, which could greatly enhance the ECL intensity. Under optimal conditions, the designed immunosensor exhibited wider linear range from 0.0001 to 30 ng/mL with a relative lower detection limit of 33 fg/mL for AFP detection. Overall, the designed immunosensor exhibited high sensitivity and selectivity, good repeatability and stability. This proposed method provided a potential application for clinical monitoring of AFP.

Alpha fetoprotein (α-fetoprotein or AFP) is an oncofetal glycoprotein produced by fetal yolk sac, which is consist of 4% carbohydrates and 591 amino acid residues^1^. It is the most reliable and important tumor markers for diagnosis of hepatocellular carcinoma (HCC)^2^,^3^ which is one of the most common cancers with high incidence and tremendous damage in the region of China and Southeast Asia as well as Africa^4^. Generally, the early diagnosis of HCC is hard to be implemented due to its asymptomatic nature, and most patients are accompanied by advanced form of the disease at diagnosis. Fortunately, the HCC growth period is strongly related with the AFP level in human serum, and the concentration of which can even monitor the occurrence of liver cirrhosis or chronic infection with hepatitis B or C virus^5^. Therefore, for the HCC patients, the early AFP detection is of crucial importance, which is helpful for the early treatment of HCC and give a higher chance of survival^6^. Given the current situation, it is essential to create a more accurate and effective method for the early detection of AFP.

Various conventional methods had been proposed for AFP detection, such as enzyme-linked immunosorbent assays (ELISA)^7^, surface plasmon resonance (SPR)^8^, fluorescence probes^9^, chemiluminescence (CL)^10^ and so forth. Recently, some faster and more convenient methods, such as electrochemistry^11^ and electrochemiluminescence (ECL)^12^,^13^, have been reported in succession. Compared with other method, ECL that assembled the advantages of various methods, such as easy operation, high sensitivity, wide dynamic range, low background signals and low-cost^14^, has attracted tremendous interests. It has been deeply studied and extensively applied to various fields including analysis of food or pharmaceutical^15^, DNA-aptamer^16^, immunoassay and so on^17^. As for the ECL system, two reaction procedures were included: the first step is the diffusion of generated reactive species from electrode. The second step is the emission of light which was induced by the reaction between electrogenerated substances and co-chemicals^18^.

However, the fixing of bioactivator may influence the immunosensor’s performance. Herein, in order to obtain better stability, the ECL and nanotechnology were combined. In the past decades, nanomaterials have been widely applied to ECL field due to their large specific surface area, high reaction activity, and good biocompatibility^19^. Among novel nanomaterials, graphene^20^, as a unique two dimensional nanomaterial, has attracted most attention due to its excellent physicochemical properties. And their application in ECL sensors is also worth noting^21^. However, a serious problem for the fabrication of graphene-based sensor is the aggregation of graphene sheets (GNSs) which is brought by the π-π interaction; it will leading to the significant loss of effective surface area and the poor ECL behavior^22^. To conquer the as mentioned problem, in this study, we employed an environmentally friendly green chemical reaction strategy to introduce carbon black (CB) into rGO. The CB shows good electrical conductivity and has attracted a wide spread attention in the development of electroanalytical sensors^23^ and other fields^24^,^25^. The use of CB was not only effectively avoided the stack and agglomeration problems of rGO, but also promoted the diffusion of electrons through rGO^26^.

Another interesting aspect of this work was the use of nanoporous metals. Recently, nanoporous metals have attracted much attention as free-standing nanomaterials in catalytic, sensing and chemical detection systems^27^ due to their superior physical and chemical properties, such as free-particle aggregation, extremely pure metal surface and excellent electron conductivity^28^. Among various nanoporous metal materials, nanoporous silver (NPS) performed much superiority benefited from its large surface-to-volume ratio, good electrical conductivity, and biocompatibility. In this study, three-dimensional (3D) bicontinuous NPS was produced by the dealloying of binary alloy^29^,^30^, which provides a new material platform for the immobilization of large numbers of Ru(bpy)_3_^2+^ and secondary antibodies (Ab_2_) acting as labels. Meanwhile, benefited from the high conductivity of silver, the electron transfer process was further promoted and then leading to the enhancement of ECL signal response.

In this work, novel sandwich-type ECL immunosensor was designed for the quantitative detection of AFP. RGO@CB functionalized with gold nanoparticles (Au NPs) and anti-AFP (Ab_1_) was used as the sensing platform. Due to the adsorption forces between gold nanoparticles (Au NPs) and rGO@CB, a mass of gold nanoparticles (Au NPs) were loaded on rGO@CB (Au-rGO@CB) to capture the anti-AFP (Ab_1_) (Au-rGO@CB-Ab_1_) via Au-NH_2_^31^. Along with the loading of Au NPs, the conductivity of composites was obviously enhanced, and which resulting the improvement of sensing sensitivity^32^. In addition, NPS, which provided a large surface area to combine with Ru(bpy)_3_^2+^ and secondary antibodies (Ab_2_) (NPS-Ru(bpy)_3_^2+^-Ab_2_), was also applied as label. Herein, Ru(bpy)_3_^2+^ was used as the luminescent reagent. And by co-reacting with triethanolamine (TEOA), strong and stable ECL signals were observed. The ECL immunosensor performed a wider linear range from 0.0001 to 30 ng/mL with a lower detection limit of 33 fg/mL for the detection of AFP. This immunosensor could be facilely constructed and had already been applied to the detection of serum samples. The result indicated that this method was practical and reliable for clinical monitoring of AFP.

## Experimental section

### Reagents and apparatus

All the starting materials and reagents were commercially purchased. Antibody of AFP and AFP were purchased from Wang Er Biochemical Reagents (Beijing, China). Sodium citrate tribasic hydrate, graphite, HAuCl_4_·4H_2_O and bovine serum albumin (BSA, 96–99%) were obtained from Sigma-Aldrich (Beijing, China). All other analytical reagents were used without further purication. 0.067 M phosphate buffered saline (PBS) was used as the electrolyte for all the electrochemistry measurements. Triethanolamine and potassium ferricyanide (K_3_[Fe(CN)_6_) were purchased from Fuyu Chemical Co., Ltd. (Tianjin, China). Ultrapure water (resistivity ≥ 18.25 MΩ cm, 25 °C) was used throughout the experiment.

Scanning electron microscope (SEM) images and energy dispersive spectrometer (EDS) were recorded using a field emission SEM (Zeiss, Germany). The ECL measurements were carried out on a MPI-F flow-injection chemiluminescence detector (Xi’an Remax Electronic Science Technology, China). Electrochemical impedance spectroscopy (EIS) measurements were performed using IM6e Electrochemical Interface (Zahner, Germany). UV-vis spectra were carried out by using a Lambda 35 UV-vis spectrometer (PerkinElmer, United States). All electrochemical measurements were performed on a CHI760D electrochemical workstation (Chenhua Instrument Shanghai, China) with a traditional three-electrode system, consisting of a platinum wire as auxiliary electrode, an Ag/AgCl electrode as reference electrode, and a glassy carbon electrode (GCE, 4 mm in diameter) as working electrode.

### Preparation of Au-rGO@CB-Ab_1_

GO was prepared from graphite according to the previously reported method with some modification^33^. Briefly, rGO@CB was synthesized as follows: 0.05 g of as-prepared GO was dispersed in 50 mL of ultrapure water and 0.025 g of CB was dissolved in 100 mL of DMF by ultrasonication for 2 h respectively at room temperature. Then 6 mL of CB dispersion (0.25 mg/mL) was added into 39 mL of DMF to form solution A, and 5 mL of GO dispersion (1 mg/mL, solution B) was added into solution A. Next, the mixture was under continuous ultrasonication for 2 h to form homogeneous solution and transferred into a volumetric flask. After shaking for 5 min, 9 μL of hydrazine hydrate (50 wt%) was added dropwise to reduce GO. Next, the reaction was heated to 100 °C and kept for 3 h in water bath, during this process, the color of the solution gradually changed from dark brown to black. Finally, the black product was isolated by vacuum filtration and washed with ethanol and ultrapure water. The ultimate product was dispersed in ultrapure water and stored at 4 °C.

Gold nanoparticles (Au NPs) were prepared according to a slightly modified literature report^34^. Gold suspension (2 mL) was added into rGO@CB solution (2 mL, 2 mg/mL) and shaking for 24 h to make abundant Au NPs load on the suface of rGO@CB, followed by centrifugation to discard redundant Au NPs. Then, the product (Au-rGO@CB) was dispersed in 2 mL of PBS (pH 7.4).

**Figure 1:**
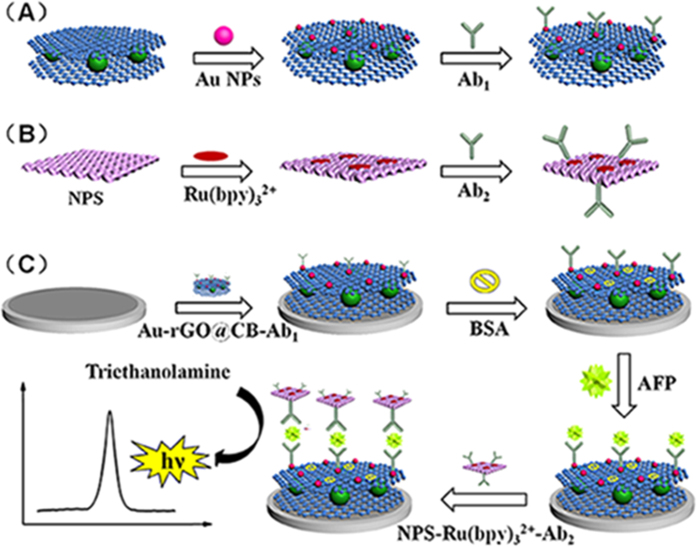
(**A**) The preparation procedure of Au-rGO@CB-Ab_1_. (**B**) The preparation procedure of NPS-Ru(bpy)_3_^2+^-Ab_2_. (**C**) The schematic illustration of the fabrication process of the sandwich-type ECL immunosensor.

Subsequently, 100 μL of Ab_1_ (10 μg/mL) and 1 mL of Au-rGO@CB were mixed and incubated for 24 h at 4 °C. Following centrifugation, the excess antibody was removed. The obtained Au-rGO@CB-Ab_1_ was dispersed in 1 mL of PBS (pH 7.4) and stored at 4 °C. The fabrication procedure of Au-rGO@CB-Ab_1_ was showed in Fig. 1A.

### Preparation of NPS-Ru(bpy)_3_^2+^-Ab_2_

NPS was prepared by a simple method^29^. Typically, Ag_10_Al_90_ alloy foils was put into 1 M NaOH solution and let stand for 12 h, followed by rinsing thoroughly with aqueous solution until neutral. The resulting NPS was dried in vacuum oven at 50 °C for 12 h.

NPS-Ru(bpy)_3_^2+^ was synthesized as following steps. First, Ru(bpy)_3_^2+^ solution (1 mM, 1 mL) was added to NPS dispersion (2 mg/mL, 1 mL) and stirred for 12 h at room temperature under lucifugal situation. Ru(bpy)_3_^2+^ could combine with NPS via electrostatic absorption. Then, the unloaded Ru(bpy)_3_^2+^ was discarded by centrifugation. Finally, the resulting NPS-Ru(bpy)_3_^2+^ was dispersed in 1 mL of PBS (pH 7.4) and stored at 4 °C.

The NPS-Ru(bpy)_3_^2+^-Ab_2_ was prepared by mixing 100 μL of Ab_2_ (10 μg/mL) and 1 mL of NPS-Ru(bpy)_3_^2+^ suspension and the mixture was incubated for 24 h at 4 °C. The obtained products were centrifuged to remove extra Ab_2_. The resulting NPS-Ru(bpy)_3_^2+^-Ab_2_ labels were dispersed in PBS (pH 7.4, 1 mL) and stored at 4 °C. Figure 1B revealed the fabrication procedure of the NPS-Ru(bpy)_3_^2+^-Ab_2_.

### Fabrication of the immunosensor

Figure 1C presented the schematic diagram of the fabrication of the immunosensor. First, glassy carbon electrode (GCE) (4 mm of diameter) was polished with alumina powder on abrasive cloth carefully and then thoroughly cleaned with ultrapure water. Then Au-rGO@CB-Ab_1_ (6 μL) was dropped on a well-polished GCE. When the electrode was dried at 4 °C, 3 μL BSA (1 wt%) was added to block nonspecific binding sites. After washing and drying, different concentrations of AFP solution (6 μL) were dropped on the electrodes, and then the electrodes were washed extensively to remove unbounded AFP molecules. Finally, the resulting electrodes were modified with as-prepared NPS-Ru(bpy)_3_^2+^-Ab_2_ (6 μL), and the modified electrodes were thoroughly washing with PBS (pH 7.4) and stored at 4 °C for further measurement.

### ECL detection of AFP

This experiment was carried out in 10 mL of PBS (pH 6.5) containing 0.1 M KCl and 0.125 M TEOA. The voltage of the photomultiplier tube (PMT) was set at 600 V and the scanning potential was from −1.8 to −0.6 V with a scan rate of 100 mV/s. Then, the modified working electrode was placed in the ECL cell to measure the ECL signal.

## Results and Discussion

### Characterization of the rGO@CB, Au-rGO@CB and NPS

**Figure 2:**
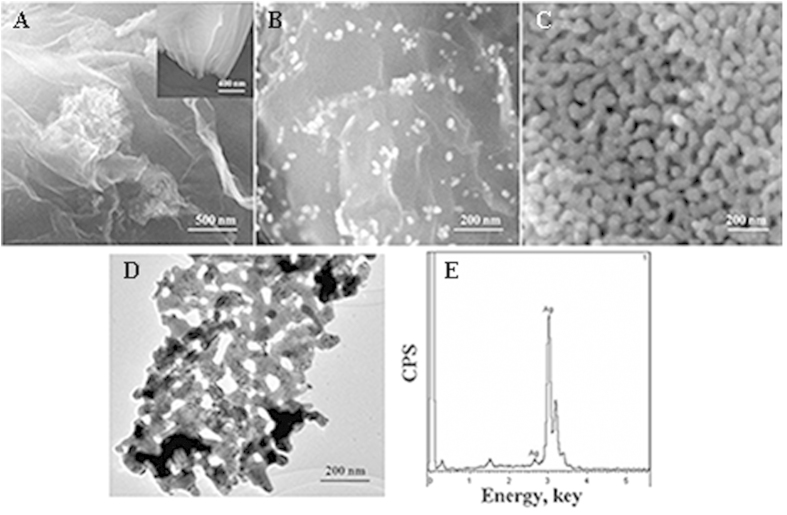
(**A**) SEM image of rGO@CB (insert is the SEM image of rGO). (**B**) SEM image of Au-rGO@CB. (**C**) SEM image of NPS. (**D**) TEM image of NPS. (**E**) EDS image of NPS.

The morphologies of rGO@CB, Au-rGO@CB and NPS were characterized by SEM. Figure 2A displayed the SEM image of rGO@CB, the obtained rGO@CB turned to plicated and sandwich structure comparing to the insert. CB as a spacer inhibited aggregation of rGO and showed a relatively stable dispersion of rGO composites. Simultaneously, it also improved the conductivity of the composites (Figure S1A).

Figure 2B showed the SEM image of Au-rGO@CB, and homogeneous distribution of Au NPs was observed on the surface of rGO@CB. Due to the large specific surface area of rGO@CB, amount of Au NPs were loaded on it, which could connect with a mount of Ab_1_ via Au-NH_2_ covalent bond^31^. Moreover, the attachment of these Au NPs could also improve the electron transfer rate. In order to further confirm that Au NPs were successfully connected on rGO@CB, the UV-vis absorbance spectrums were investigated (Figure S1B). The absorption peak of pure Au NPs was at approximately 519 nm (curve b) and there is no obvious absorption peak observed from rGO@CB (curve a). When Au NPs connected with rGO@CB, a major absorption peak around 519 nm was observed again (curve c), which indicated the successful fabrication of Au-rGO@CB.

Figure 2C displayed the SEM image of NPS, and a three-dimensional continuous interpenetrating ligament-channel porous structure could be clearly seen in the NPS sample. TEM image in Fig. 2D further verifies the porous structure of NPS with the ligaments/pores. The EDS image of NPS was shown in Fig. 2E. The EDS result confirmed that Al was nearly corroded by NaOH. N_2_-adsorption-desorption isotherm was employed to analyze the size distribution of pores (Figure S2). The plot of the pore size distribution was determined using Barrett-Joyner-Halenda (BJH) method from the adsorption branch of the isotherm. And the pore size of the synthesized NPS is approximate 13.96 nm.

### Characterization of the NPS-Ru(bpy)_3_^2+^-Ab_2_ labels

The UV-vis absorbance spectra of NPS, Ru(bpy)_3_^2+^ and NPS-Ru(bpy)_3_^2+^ were explored to testify the well combination of NPS and Ru(bpy)_3_^2+^. From Figure S3 (curve a), it was evident that the characteristic absorption peak of NPS was at approximately 400 nm. The prominent broad absorption band of Ru(bpy)_3_^2+^ (curve b) was observed at about 453 nm due to the spin allowed dπ(Ru)-π(ligand)* metal-to-ligand charge transfer (MLCT) transitions. In addition, owing to the π-π* electron transfers of pyridine ring, there was a characteristic absorption peak at about 286 nm^35^. Moreover, the characteristic absorption peaks of NPS and Ru(bpy)_3_^2+^ were also found from curve c, indicating that Ru(bpy)_3_^2+^ has been loaded on NPS successfully. Simultaneously, the zeta potential of NPS dispersion was measured at −1.36 V by Malvern zetameter (Zetasizer 2000), which revealed that the as-prepared NPS attained a negatively charged surface^36^. After the uptake of Ru(bpy)_3_^2+^, the zeta potential of composites remained smaller negative with −0.0568 V, which further manifested Ru(bpy)_3_^2+^ could well attach on the surface of NPS by electrostatic absorption.

**Figure 3:**
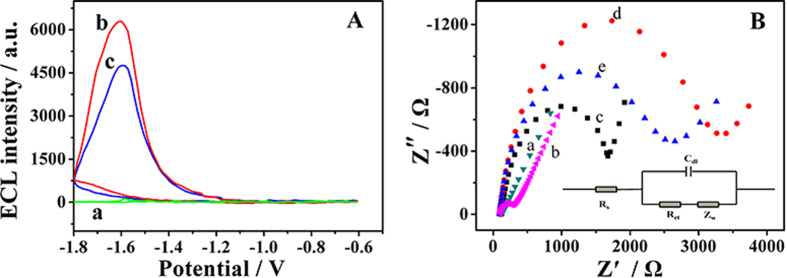
(**A**) ECL-potential curves of (a) NPS, (b) NPS-Ru(bpy)_3_^2+^, (c) NPS-Ru(bpy)_3_^2+^-Ab_2_ modified glass carbon electrode in PBS (pH 6.5) containing 0.1 M KCl and 0.125 M TEOA. Scan rate: 100 mV/s, the voltage of the PMT was 600 V. (**B**) EIS of 2.5 mM [Fe(CN)_6_]^3/4−^ at different stages: (a) bare GCE, (b) GCE/Au-rGO@CB-Ab_1_, (c) GCE/Au-rGO@CB-Ab_1_/BSA, (d) GCE/Au-rGO@CB-Ab_1_/BSA/Ag, (e) GCE/Au-rGO@CB-Ab_1_/BSA/Ag/NPS-Ru(bpy)_3_^2+^-Ab_2_, the inset is equivalent circuit for EIS.

Next, in order to certify the successful conjugation of Ab_2_ and NPS-Ru(bpy)_3_^2+^, the corresponding ECL-potential profiles of the immobilization process of NPS-Ru(bpy)_3_^2+^-Ab_2_ labels were shown in Fig. 3A, which carried out in pH 6.5 PBS containing 0.125 M TEOA. When the pure NPS was tested, there was almost no ECL signal (curve a). After NPS was coated with Ru(bpy)_3_^2+^, a remarkable ECL increase was observed. By contrast, as Ab_2_ was combined on NPS-Ru(bpy)_3_^2+^, the ECL intensity was weakened significantly (curve c). This is because Ab_2_ is a type of protein which could hinder the electron transfer between TEOA and luminescent reagents. It was demonstrated that NPS-Ru(bpy)_3_^2+^-Ab_2_ labels was successfully fabricated.

### Characterization of the immunosensor

In order to monitor the assembly of the immunosensor step by step, EIS was recorded in 2.5 mM [Fe(CN)_6_]^3/4−^ solution, which was used to studied the interfacial property of the modified electron. Figure 3B showed the result of EIS at different modification stages. The insert of Fig. 3B shows the equivalent circuit for EIS containing the bulk solution resistance (*R*_s_), the electron-transfer resistance (*R*_et_), the Warburg element (*Z*_W_) and the charge of the constant phase element (*C*_dl_). The *R*_s_ is the semicircle diameter at higher frequencies corresponding to the *R*_et_, and the linear part at lower frequencies corresponds to the diffusion process^37^.

Concretely, it was evident that the EIS of the bare GCE presented almost a straight line (curve a), which demonstrated the bare GCE existing the low electron transfer resistance. After modified with Au-rGO@CB-Ab_1_, it was observed that a relative smaller resistance (curve b) was appeared which attributing to the good electron transfer ability of Au-rGO@CB. Subsequently, with the modification of BSA (curve c) and AFP (curve d) layer by layer, the diameter of semicircle portion at high frequencies increased gradually due to the resistance of non-conductive bioactive substances. When NPS-Ru(bpy)_3_^2+^-Ab_2_ (curve e) was dropped on the electrode, the diameter of semicircular in EIS further decreased. The reason was that NPS possessed excellent electrical conductivity, which resulted in the further boost of the electron transfer ability. As the consequence, the change of the electron transfer resistance monitored the modified materials successfully assembling onto the electrode surface.

### Optimization of experimental conditions

**Figure 4:**
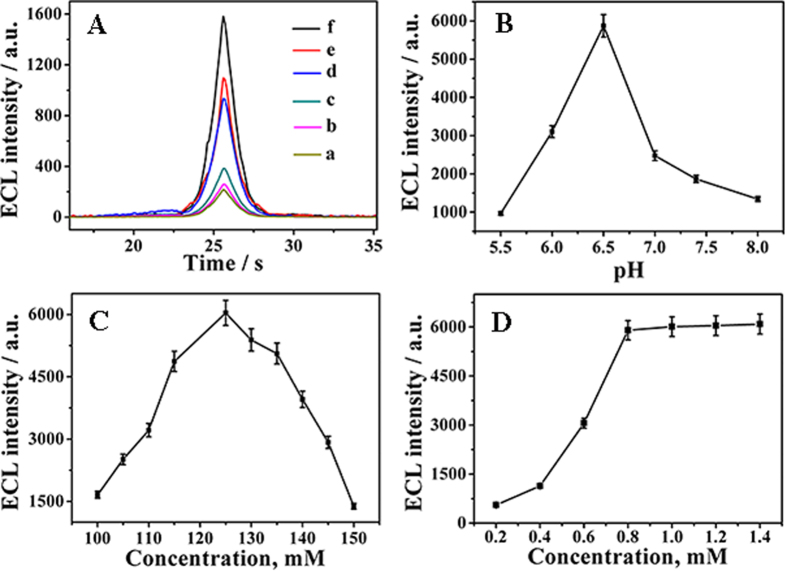
(**A**) ECL responses of immunosensor for the detection of 10 ng/mL of AFP with different sensing platform including CB (a), rGO (b), rGO@CB (c), Au-CB(d), Au-rGO (e) and Au-rGO@CB (f). Optimization of the experimental conditions with pH (**B**), TEOA concentration (**C**) and Ru(bpy)_3_^2+^ concentration (**D**). (Error bar = SD, n = 3).

In order to demonstrate the improved performance of Au-rGO@CB for the signal amplification in the designed immunosensor, control assays for the detection of 10 ng/mL of AFP (Fig. 4A) were carried out by using different sensing platforms, including CB (curve a), rGO (curve b), rGO@CB (curve c), Au-CB(curve d), Au-rGO (curve e) and Au-rGO@CB (curve f). Obviously, it was observed from these experiments that the ECL response of using rGO@CB as sensing platform to build the immunosensor was relatively greater than free-standing materials, which might be ascribed that the introduce of CB could prevent rGO from accumulating and improve the conductivity of substrate material. However, bare CB and rGO could only provide scarce sites for the combination of antibody, the ECL intensity of which was low. By contrast, the immunosensor using Au NPs functional-substrate material exhibited greater ECL response, which might be ascribed to the following reasons: Au NPs could provide a friendly microenvironment to maintain the biocompatibility of immobilized biomolecules^38^ and also acted as the conducting tunnel to promote the electron transfer, increasing the sensitivity of biosensor^39^. The result showed that employment of Au-rGO@CB could improve the performance of immunosensor.

Moreover, for the sake of maximal ECL signal, the optimization of experimental parameters was investigated, including pH value of PBS, TEOA concentration, the loading amount of Ru(bpy)_3_^2+^, Ab_1_ concentration and Ab_2_ concentration. As shown in Fig. 4B, ECL intensity increased with the pH value from 5.5 to 6.5 and then decreased in the range of 6.5 to 8.0. This might ascribed that under the overly acidic or alkaline surroundings, especially in alkalinity, protein would denature and lose their effectiveness to immobilize on the nanometerial^40^. Thus the optimal pH value was selected as 6.5.

Besides, the co-reactant TEOA concentration was an important factor impacting the immunosensor signal. As Fig. 4C displayed, the ECL signal increased with TEOA concentration in the range of 0.1 to 0.125 M because more Ru(bpy)_3_^2+*^ was generated. When TEOA concentration is higher than 0.125 M, the ECL signal decreased which was ascribed to that the excess TEOA with the poor conductivity would hinder the electron transfer in solution. Therefore, the TEOA concentration of 0.125 M was used in this work.

The immobilization amount of Ab_1_ on the sensing platform has a great important effect on the ECL signal. So, the immobilization amount of Ab_1_ was investigated. The results were shown in Figure S4A. When the concentration of Ab_1_ was more than 10 μg/mL, the ECL response nearly achieved a platform value, so the optimal incubation concentration of Ab_1_ was selected as 10 μg/mL.

What is more, the loading amount of Ru(bpy)_3_^2+^ also has a great influence on the ECL signal readout. Therefore, a series of concentration of Ru(bpy)_3_^2+^ from 0.2 to 1.4 mM were selected. As shown in Fig. 4D, the ECL intensity increased with the increase of the concentration from 0.2 to 0.8 mM in large amplitude. After more than 0.8 mM, the ECL signal rose slowly up the concentration of Ru(bpy)_3_^2+^, indicating that the loading amount of Ru(bpy)_3_^2+^ has reached saturated basically on the surface of NPS. For sake of sufficient combination of NPS and Ru(bpy)_3_^2+^, 1.0 mM was chosen as the optimal concentration of Ru(bpy)_3_^2+^ for subsequent analytical experiments. Redundant Ru(bpy)_3_^2+^ could be removed by centrifugation.

For the sandwich-type immunosensor, the amount of Ab_2_ on the labels has a great important effect on the ECL signal. Thus, the effect of concentration of Ab_2_ was investigated from 2 to 14 μg/mL. As shown in Figure S4B, the ECL intensity increased with the increase of the concentration from 2 to 10 μg /mL. After more than 10 μg/mL, the ECL response achieved a platform value due to the saturated loading of Ab_2_ on the surface of NPS. So, 10 μg/mL was selected as the optimum concentration of Ab_2_.

### ECL behavior of AFP with the immunosensor

**Figure 5:**
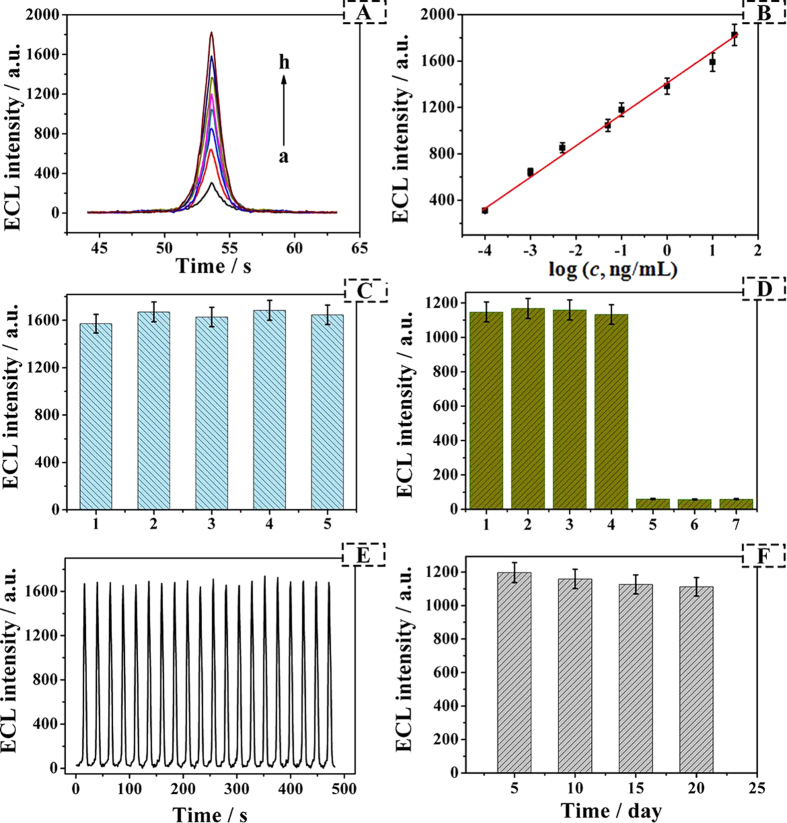
(**A**) The ECL response of the immunosensor to different concentrations of AFP, from a to h: 0.0001, 0.001, 0.005, 0.05, 0.1, 1, 10, 30 ng/mL. (**B**) Calibration curve of the immunosensor for AFP detection at different concentrations. (**C**) The repeatability of the GCE/Au-rGO@CB-Ab_1_/BSA/Ag/NPS-Ru(bpy)_3_^2+^-Ab_2_ biosensor in PBS (pH 6.5) containing 0.1 M KCl and 10 ng/mL AFP. (**D**) The effect of interferents for ECL intensity: (1) 0.1 ng/mL AFP; (2) 0.1 ng/mL AFP and 10 ng/mL carcinoembryonic antigen (CEA); (3) 0.1 ng/mL AFP and 10 ng/mL BSA; (4) 0.1 ng/mL AFP and 10 ng/mL glucose; (5) 0.1 ng/mL CEA; (6) 0.1 ng/mL BSA; (7) 0.1 ng/mL glucose. (Error bar = SD, n = 3). (**E**) Stability of ECL sensor incubated with AFP (10 ng/mL) under continuous scanning for 20 cycles in PBS (pH 6.5) containing 0.1 M KCl and 0.125 M TEOA. (**F**) The long-term storage stability of the biosensor incubated with 0.1 ng/mL AFP measuring in PBS (pH 6.5) containing 0.1 M KCl and 0.125 M TEOA.

The proposed immunosensor with different concentrations of AFP standard solution was investigated under the optimal condition (Fig. 5A). As expected, the ECL intensity increased with the increasing concentration of AFP. It was observed in Fig. 5B that the ECL intensity increased linearly with the logarithm of AFP concentration from 0.0001 to 30 ng/mL. The regression equation of the calibration curve was *I*_ECL_ = 1399.270 + 259.666 lg [*c*] (R = 0.996). The limit of detection (LOD) of this work was 33 fg/mL (S/N = 3). In addition, there was a comparison with different methods of AFP detection in table S1. Compared with LOD of ELISA (1 ng/mL)^7^, SPR (0.65 pg/mL)^8^, fluorescence (0.28 pg/mL)^9^, chemiluminescent (0.52 pg/mL)^10^, electrochemistry (0.01 ng/mL)^41^ and even ECL (0.2 pg/mL)^12^, the proposed immunosensor presented a relative lower LOD for the determination of AFP. The lower LOD might be ascribed two factors: (1) high electron transfer efficiency and excellent conductivity of Au-rGO@CB could greatly enhance the ECL response. (2) The NPS with large specific surface area could fix a large amount of Ru(bpy)_3_^2+^ and Ab_2_, which exhibit a better ECL signal. In general, the scheme might be a feasible way to detect AFP.

### Repeatability, Selectivity, and stability

To explore the repeatability of the fabricated ECL sensor, a series of five electrodes were modified with 10 ng/mL of AFP on the same condition (Fig. 5C). The relative standard deviation (RSD) of the measurements for the five electrodes was 1.13%. The results indicated that the immunosensor had satisfied repeatability.

The selectivity of the fabricated ECL sensor was investigated. The as-prepared electrode was incubated with 0.1 ng/mL of AFP solution containing different interfering substances at a concentration of 100 times higher than that of AFP, such as carcinoembryonic antigen (CEA), BSA and glucose. As could be seen from Fig. 5D, the ECL response of the proposed immunosensor showed negligible change in the AFP mixed with interfering agents compared to AFP only with the RSD of 1.70%. On the contrary, weak ECL responses were exhibited by replacing AFP with CEA, BSA and glucose. The result manifested that the selectivity of the immunosensor was great.

Simultaneously, operational stability and the long-term storage stability were vital factors to evaluate availability of the immunosensor. As the electrode modified with 10 ng/mL AFP was scanned from −1.8 to −0.6 V in PBS (pH 6.5) containing 0.1 M KCl and 0.125 M TEOA at 0.1 V/s for 20 cycles (Fig. 5E), the stable ECL signal was observed with the RSD of 0.72%. In addition, the long-term storage stability was investigated at 0.1 ng/mL AFP solution by storing the biosensor at 4 °C and testing every five days (Fig. 5F). After 20 days, the ECL intensity of the biosensor was remained approximately 92.9% of the original value. The above results testified that the biosensor has excellent stability.

### Application for AFP samples

To verify the precision and accuracy of this designed immunosensor, the test with different concentrations of AFP in human serum was carried out using standard addition method^42^. As shown in table S2, the corresponding RSD was in the range of 1.06% to 5.35% and the recovery of the proposed sensor was in range from 95.3% to 101%. The result demonstrated that as-fabricated method provided a simple and applicable way for the quantitative detection of AFP in real samples with acceptable and satisfactory result and would have potential application for highly sensitive clinical analysis.

## Conclusion

In this study, a novel and ultrasensitive sandwich-type ECL immunosensor has been created for the detection of AFP, which applied Au-rGO@CB as sensing platform and NPS-Ru(bpy)_3_^2+^ as labels. The conductivity of the substrate material was improved due to the insertion of carbon black and the decoration of Au NPs. Meanwhile, NPS with abundant pore structure provided more binding sites for Ru(bpy)_3_^2+^ and Ab_2_, which made the immunosensor exhibit stronger ECL signal in the presence of TEOA. This simple immune method for detection of AFP would hold enormous promise in clinical diagnosis.

## Additional Information

**How to cite this article**: Zhu, W. *et al.* Ru(bpy)_3_^2+^/nanoporous silver-based electrochemiluminescence immunosensor for alpha fetoprotein enhanced by gold nanoparticles decorated black carbon intercalated reduced graphene oxide. *Sci. Rep.* **6**, 20348; doi: 10.1038/srep20348 (2016).

## Supplementary Material

Supplementary Information
