# Supplementary material for: Ru(bpy)32+/nanoporous silver-based electrochemiluminescence immunosensor for alpha fetoprotein enhanced by gold nanoparticles decorated black carbon intercalated reduced graphene oxide
Source: Sci Rep. 2016 Feb 1;6:20348. doi: 10.1038/srep20348 (PMC4734325; doi:10.1038/srep20348)
Supplement: Supplementary Information [file srep20348-s1.pdf]

Supporting Information

$Ru(bpy)_3^{2+}$/nanoporous silver-based electrochemiluminescence immunosensor for alpha fetoprotein enhanced by gold nanoparticles decorated black carbon intercalated reduced graphene oxide

Wenjuan Zhu[a], Xiaohui Lv[a], Qi Wang[b], Hongmin Ma[a], Dan Wu[a], Tao Yan[a], Lihua Hu[a], Bin Du[a], Qin Wei[a]*

[a]*Key Laboratory of Chemical Sensing & Analysis in Universities of Shandong, School of Chemistry and Chemical Engineering, University of Jinan, Jinan 250022, P.R. China*

[b]*School of Material Science and Engineering, University of Jinan, Jinan 250022, P.R. China*

* **Corresponding author.**

Tel: +86 531 82767872;

Fax: +86 531 82767367

E-mail address: sdjndxwq@163.com (Q. Wei)

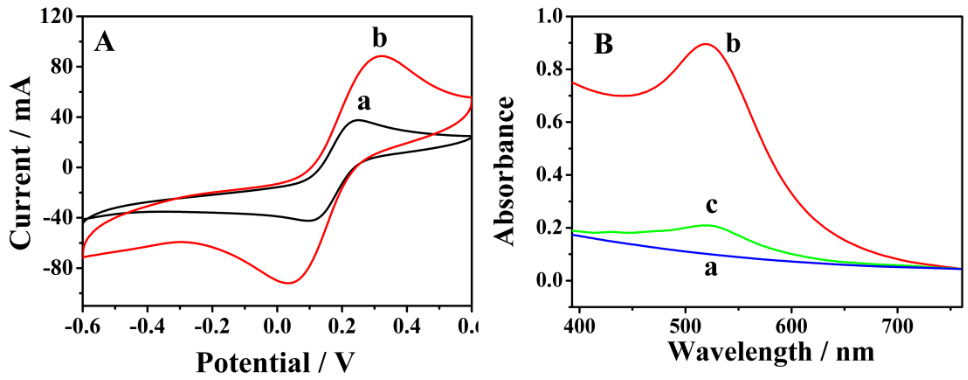

Figure S1. (A) CVs recorded from -0.6 to 0.6V: rGO/GCE (a) and rGO@CB/GCE (b). (B) UV-vis absorption spectra of rGO@CB (a), Au NPs (b), Au-rGO@CB (c).

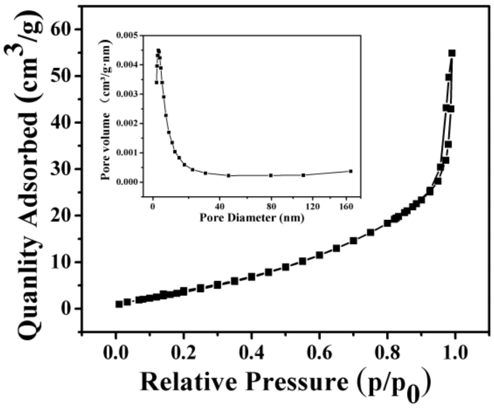

Figure S**2.** BET analysis results of NPS.

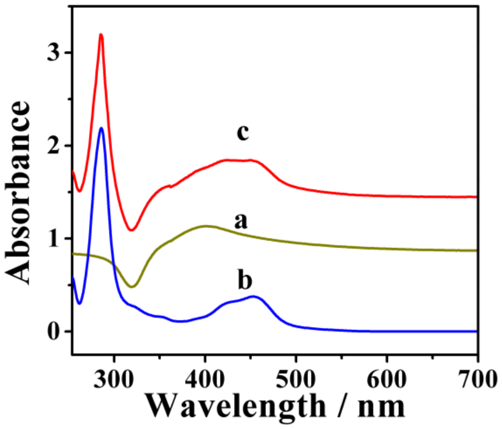

Figure S3. The UV-Vis absorbance spectra of NPS (a), $Ru(bpy)_3^{2+}$ (b) and NPS-$Ru(bpy)_3^{2+}$ (c).

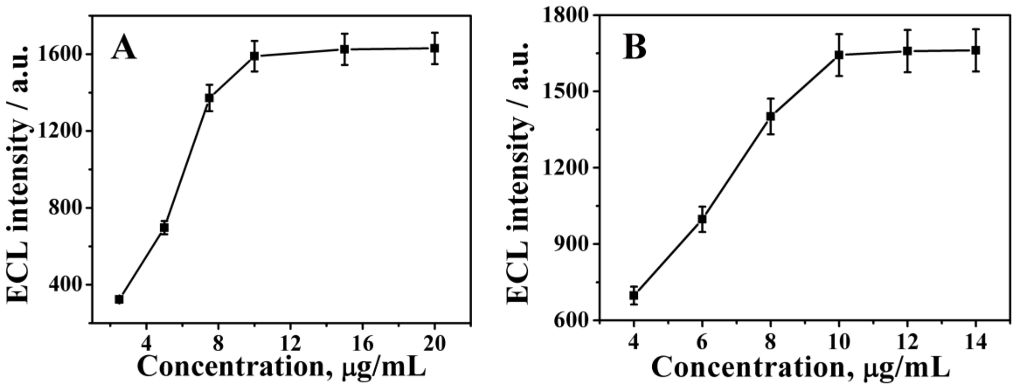

Figure S4. Optimization of the experimental conditions with Ab_1 concentration (A) and Ab_2 concentration (B). (Error bar = SD, n = 3).

Table S1. A comparison with other methods for the detection of AFP

Method	Material of sensor	Linear range (ng/mL)	LOD (pg/mL)	Reference
ELISA	PEI-modified PMMA Microchannels	0.001-0.5	1	7
SPR	MPS/CS-Fc/Au NPs	0.001-0.2	0.65	8
Fluorescence	multicolor quantum dots	0.5-500	0.28	9
CL	biofunctionalized-MWCNTs	0.001-0.1	0.52	10
Electrochemistry	graphene/SnO_2/Au nanocomposite	0.02-50	10	41
ECL	Fe_3O_4-Au magnetic nano probes and CdS-Au	0.0005-5	0.2	12
ECL	Au-rGO@CB and NPS	0.0001-30	0.033	This work

Table S2. Results of AFP in human serum sample by the ECL immunosensor

Original（ng/mL）	Addition (ng/mL)	Detection amounts（ng/mL）	Average value（ng/mL）	RSD（%）	Recovery（%）
	1.00	1.72, 1.87, 1.76, 1.97, 1.85	1.83	5.35	95.3
0.92	3.00	3.99, 3.78, 3.93, 3.82, 3.89	3.88	2.17	98.9
	5.00	5.84, 5.93, 5.76, 5.89, 5.97	5.97	1.06	100.8
